# Meteorological Journal

**Published:** 1831-02

**Authors:** 


					186
METEOROLOGICAL JOURNAL,
By Messrs. Harris and Co. Mathematical Instrument Makers,
50, High Holborn.
Dec
20
21
22
23
24
25
20
27
28
29
30
31
Jan.
1
2
3
4
5
6
7
8
9
10
11
12
13
14
15
16
17
18
19
o
*
Ob
.03
Therm
Barom.
29.34
.71
.41
.29
.28
.17
.17
.03
28.95
29.54
.28
.08
.65
.72
.76
.72
.68
30.07
.42
.42
.06
29.74
.94
.92
.99
30.03
29.80
.63
.45
.40
.50
29.52
.63
.23
.39
.16
.17
.17
.04
.26
.56
.06
.41
.72
.75
.76
.69
.79
30.25
.42
.31
29.83
.81
.94
.92
.99
30.00
29.72
.51
.43
.43
.48
De Luc's
Hygrom
Winds.
NNW
N
w
NW
NW
W
W
SE
SE
WSW
E Vtt.
S
WSW
WSW
s
SE
E
N
NNE
WNW
NW
NNE
E
ENE
NE
ENE
SE
SE
SE
ESE
SSW
N
WSW
NW
NNE
W
W
NNE
E
NW
SSW
8
WNW
SW
WSW
s va.
ESE
NE
NNE
NNE
W
SW
ENE
E
E
NE
SSE
S
SE
ESE
8
Atmospheric
Variation.
Fine
Cloud)
Foggy
Fine
Foggy
Foggy
Foggy
Cloudy
Fine
Cloudy
Fog
Foggy
Fine
Foggy
Cloud)
Fine
Foggy
Cloudy
Foggy
F.&S1.
Fine
Rain
Fine
Fine
Fine
Foggy
F.?feSl,
Fine
Fine
Foggy
Fine
Cloudy
Fine
Cloud)
Cloudy
Foggy
Cloudy
Foggy
Cloudy
Foggy
Sleet
S
Fine
Fine
Foggy
Cloudy
Cloudy
Fine
Cloudy
Fine
Cloudy
Foggy
Cloudy
Fine
Fine
Sleet
Cloudy
Cloudy
Cloudy
Cloudy
Foggy
Cloudy
The Rain Gauge having burst, from the late frost, the quantity of Rain
cannot be given.

				

